# Retraction Note: Factors associated with insomnia among frontline nurses during COVID-19: a cross-sectional survey study

**DOI:** 10.1186/s12888-022-04130-8

**Published:** 2022-07-25

**Authors:** Nabi Nazari, Masoud Sadeghi, Vadim Samusenkov, Akram Aligholipour

**Affiliations:** 1 Department of Psychology, Faculty of Human Sciences, Lorestan University, Khorramabad, Iran; 2 Department of Prosthetic Dentistry, Sechenov First Moscow State Medical University, Moscow, Russia; 3 Departments of Psychology, Faculty of Human Sciences, Allameh Tabatabai University, Tehran, Iran

**Retraction Note: BMC Psychiatry 22, 40 (2022)**

**https://doi.org/10.1186/s12888-022-03690-z**

The Editor has retracted this article. After publication concerns were raised that numerous statements in the article are not supported by the references cited. The authors have not provided a satisfactory explanation for these concerns. In addition, the authorship contribution has not been verified. The Editor therefore no longer has confidence in the veracity of the work presented. Nabi Nazari and Vadim Samusenkov disagree with this retraction. Masoud Sadeghi and Akram Aligholipour have not responded to correspondence from the Editor about this retraction.

